# Dataset on effect of cadmium and lead on chemical reactivity of selected heterocyclic compounds as potential dipeptidyl peptidase III inhibitors using *insilico* method

**DOI:** 10.1016/j.dib.2025.111481

**Published:** 2025-03-20

**Authors:** Kehinde Adeola Bolaji, Abel Kolawole Oyebamiji, Godwin Oladele Olutona

**Affiliations:** a Industrial Chemistry Programme, Bowen University, PMB 284, Iwo, Osun State, Nigeria; b Department of Physical Science Education, Emmanuel Alayande University of Education, Oyo, Oyo State, Nigeria; c Department of Industrial Chemistry, University of Ilesa, Osun State, Nigeria; d Good Health and Wellbeing Research Clusters (SDG 03), University of Ilesa, Ilesa, Osun State, Nigeria

**Keywords:** Lead, Cadmium, Heterocycles, Docking, Dipeptidyl peptidase III

## Abstract

The role played by heterocyclic compounds in drug design and discovery remain crucial globally. In this work, the effect of cadmium and lead were investigated on the chemical reactivity of selected heterocyclic compounds with potential anti-dipeptidyl peptidase III activity using *insilico* method. Various software such as Spartan 14 (for optimization), molecular operating environment (moe) (for induced fit docking) and ADMETSar (pharmacokinetic examination) were used to execute the investigations. Three descriptors (highest occupied molecular orbital energy (EHOMO); lowest unoccupied molecular orbital energy (ELUMO) and energy gap) were observed among others for selected compounds without any foreign attachment, selected compounds with cadmium and selected compounds with lead. The calculated descriptors revealed that cadmium had a great effect on compound **7** in term of HOMO energy and energy gap. Also, it was observed that cadmium altered the ability of compound 5 in term LUMO energy. The selected compounds were docked against dipeptidyl peptidase III and the scoring was reported. More so, pharmacokinetic examination of ligands with highest binding affinity was reported and compared to the report for the reference molecules.

Specifications Table

**Table:** 

Subject	*Insilico* Study
Specific subject area	Drug Evaluation
Type of data	Figure, Chart, Table, ADMET, Graph
Data collection	The investigated compounds (compound without foreign atom; compound + cadmium and compound + lead) were theoretically calculated using Spartan 14 in order to have access to the features encoded in the examined compounds. The compounds with no foreign atom attached were docked into the active site of Dipeptidyl Peptidase III [pdb id: 3fvy] using induced fit method via molecular operating environment software. The role of retrieved descriptors from theoretically calculated compounds in the inhibiting ability of the examined compounds were investigated using Microsoft excel software and pharmacokinetics evaluation was exhibited on compound with highest binding affinity. Every outcome was reported and described.
Data source location	Computational Chemistry Research Laboratory, Department of Chemistry and Industrial Chemistry, BOWEN University, PMB 284, Iwo, Osun State, Nigeria.
Data accessibility	https://data.mendeley.com/datasets/wrhd27phzc/1 Repository name: Mendeley Data Data identification number: 10.17632/wrhd27phzc.1 Direct URL to data: https://data.mendeley.com/datasets/wrhd27phzc/1 Instructions for accessing these data: The data can be accessed using the above URL
Related research article	*None*

## 1 Value of the Data

• The calculated descriptors will show to scientists the effect of cadmium and lead on the geometries of the compounds under study.
• The two dimensional structure of the examined compounds will expose to researchers the nature of the bond between atoms used in the work.
• The configuration of the raw and treated receptors will be exposed to researchers.
• The calculated features for individual compound will reveal to researchers the type of reaction the examined compound can be involved in.
• The scoring, the calculated distance, the predicted bond type and amino acid residue will show to scientists the nature of interactions involved in the examined complexes.
• The results of the pharmacokinetic analysis will provide information on the absorption, digestion, metabolism, and excretion processes of the molecule with the highest binding affinity in the human system.

## 2 Background

The objectives of this work are:

➢ To investigate the biological effects of cadmium and lead on geometries (bond length and bond angle) of the optimized selected heterocyclic compounds.
➢ To evaluate the biochemical efficiency of the optimized selected heterocyclic compounds docked against dipeptidyl peptidase III [pdb id: 3fvy] in order to down-regulate pain in mammals.
➢ To identify the ability of the compound with highest binding affinity to act as drug-agent via pharmacokinetics evaluation.

## 3 Data Description

The 2-dimensional and 3-dimenional structure of the examined compound as well as the IUPAC name of the examined compound were presented on Table 1. As shown in Table 1, the 3-dimensional structure of the examined compounds comprises of atom which were represented by using different ball (red=oxygen; blue=Nitrogen; yellow = Sulphur) were bonded together to form the compounds under investigation.

**Table 1:** 2-Dimensional and 3-dimensional Structures and IUPAC name of the Investigated compounds.

	2-D Structure	IUPAC Names
1		2-acetoxybenzoic acid
2		3-ethyl 5-methyl 2-((2-aminoethoxy)methyl)-4-(2-chlorophenyl)-6-methyl-1,4-dihydropyridine-3,5-dicarboxylate
3		N4-(7-chloroquinolin-4-yl)-N1,N1-diethylpentane-1,4-diamine
4		3-(4-chlorophenyl)-N,N-dimethyl-3-(pyridin-2-yl)propan-1-amine
5		1-cyclopropyl-6-fluoro-4-oxo-7-(piperazin-1-yl)-1,4-dihydroquinoline-3-carboxylic acid
6		1-((2-chlorophenyl)diphenylmethyl)-1H-imidazole
7		2-(2-methyl-5-nitro-1H-imidazol-1-yl)ethan-1-ol
8		nicotinamide
9		N-(4-hydroxyphenyl)acetamide
10		4-hydroxy-2-methyl-N-(pyridin-2-yl)-2H-benzo[e][1,2]thiazine-3-carboxamide 1,1-dioxide

Table 2 showed the calculated features (highest occupied molecular orbital energy (EHOMO), lowest unoccupied molecular orbital energy (ELUMO) and energy gap) for the optimized compound, optimized compound + cadmium and optimized compound + lead. The graphical representation of the HOMO and LUMO orbital energy profile for the entire compound investigated in this work was displayed in Table 3.

**Table 2:** Selected features for the optimized compounds.

	Selected Compound			Selected Compound + Cadmium			Selected Compound + Lead		
	HOMO (eV)	LUMO (eV)	Energy Gap (eV)	HOMO (eV)	LUMO (eV)	Energy Gap (eV)	HOMO (eV)	LUMO (eV)	Energy Gap (eV)
1	-10.27	-1.20	9.07	-7.23	-2.53	4.70	-10.28	-1.22	9.06
2	-8.75	-0.47	8.28	-7.56	-2.76	4.80	-8.78	-0.49	8.29
3	-8.55	-0.95	7.60	-7.39	-2.59	4.80	-8.55	-0.95	7.60
4	-8.35	-0.22	8.13	-7.48	-2.70	4.78	-8.36	-0.24	8.12
5	-9.01	-0.99	8.02	-6.41	-4.57	1.84	-9.01	-0.99	8.02
6	-9.22	-0.49	8.73	-7.70	-2.89	4.81	-9.23	-0.49	8.74
7	-10.65	-2.04	8.61	-6.03	-4.26	1.77	-10.63	-2.06	8.57
8	-10.52	-0.89	9.63	-7.68	-2.89	4.79	-10.51	-0.88	9.63
9	-9.17	-0.06	9.11	-7.83	-3.02	4.81	-9.17	-0.05	9.12
10	-9.43	-1.49	7.94	-7.83	-3.02	4.81	-9.43	-1.50	7.93

**Table 3:** Predicted orbital energy profile for the selected compounds.

	Selected Compound	Selected Compound + Cadmium	Selected Compound + Lead
**1**			
**2**			
**3**			
**4**			
**5**			
**6**			
**7**			
**8**			
**9**			
**10**			

**Fig. 1:**
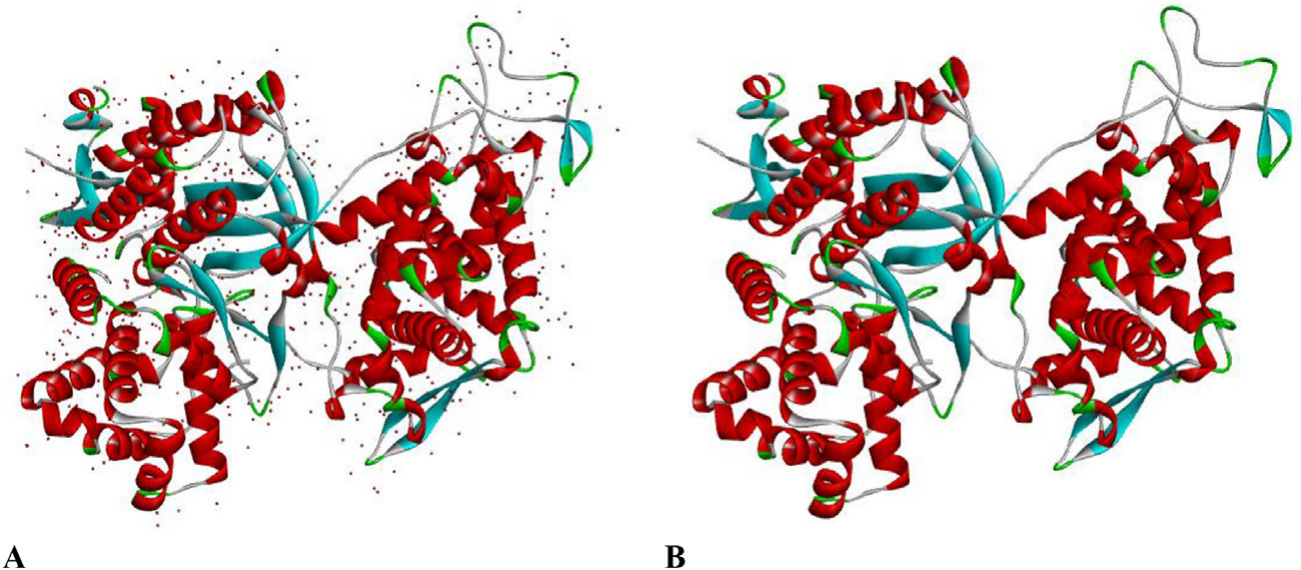
3-dimensional structure of (A) raw Dipeptidyl Peptidase III (B) treated Dipeptidyl Peptidase III.

Fig. 1 revealed raw Dipeptidyl Peptidase III and treated Dipeptidyl Peptidase III. Fig. 1A showed the investigated receptor with water molecules and other foreign small molecules while Fig. 1B showed the cleaned Dipeptidyl Peptidase III. Also, Table 4 showed the predicted binding sites obtained from the treated Dipeptidyl Peptidase III. The factors considered were size, protein ligand-binding (PLB), Hyd, side and residues.

**Table 4:** The calculated binding site for dipeptidyl peptidase III [pdb id: 3fvy].

Site	Size	PLB	Hyd	Side	Residues
1	544	4.76	134	264	1:(GLN5 ILE7 LEU8 PRO9 ASP11 ILE12 GLY13 ASP18 CYS19 ARG20 GLU21 ALA22 TRP42 VAL48 TYR100 SER101 ASN102 MET103 SER108 PHE109 GLY110 ASP111 TYR171 GLU316 SER317 TYR318 ARG319 GLU327 PHE328 GLU329 VAL334 VAL335 ASN336 MET339 SER340 LYS342 PHE343 ASP372 PHE373 THR374 SER375 LEU376 ASP377 PHE381 ALA382 GLY383 SER384 GLY385 ILE386 PRO387 ALA388 ASN406 VAL4 ... AL673 GLN674 LEU696 SER699 PHE700 ARG703)
2	47	0.13	18	45	1:(TYR35 SER38 ARG39 TRP42 THR380 PRO675 ASN676 THR677 ARG678 LEU679 GLU702 ARG703 PHE704 PRO705 GLU706)
3	85	0.07	27	54	1:(TYR58 ALA61 ARG65 ARG68 ALA69 ALA132 GLN135 HIS136 GLU139 VAL140 GLY142 LEU143 THR146 PRO709 GLU712 GLU713 THR716)
4	50	0.05	19	33	1:(TYR60 LEU63 SER64 PHE67 ARG68 TYR100 ILE697 PHE700 SER701 GLU702 ARG703 PHE704 PRO705 GLU706 ASP707 GLY708 PRO709 GLU712)
5	37	-0.11	4	19	1:(ARG421 GLU422 LYS423 LEU424 THR425 PHE426 LEU427 GLU429 LYS432 ASP600 ALA601 ARG602 ARG665)
6	35	-0.18	15	29	1:(ASN192 LEU193 SER194 ASN197 LEU216 GLU229 VAL230 LYS233 GLY288 SER289 ILE290 GLU291 LYS294)
7	11	-0.19	7	31	1:(TYR35 HIS36 ARG39 TYR43 LYS257 GLU260 GLN261 LYS264 LEU679 GLU706 ASP707)
8	10	-0.21	5	15	1:(ARG215 ARG248 GLY249 ASP250 TYR251 LEU718 ALA721 ASP722)
9	45	-0.21	19	29	1:(ASP303 LYS304 GLY305 PRO306 GLU309 VAL335 ASP372 THR374 LEU376 PHE404 LYS405 ASN406 VAL407)
10	39	-0.22	10	22	1:(THR501 ILE502 SER505 TYR557 ALA561 PHE562 ASN563 TRP564 GLN570 GLU642 ALA645 THR646)
11	23	-0.22	9	21	1:(SER194 TYR196 ILE290 LYS294 ASN394 TYR395 ASP396 ASP397)
12	31	-0.22	13	21	1:(GLU351 LEU354 LYS355 GLU356 LEU357 PRO358 TRP359 PRO360 PRO361 GLU364 LYS365 ASP366)
13	21	-0.23	7	18	1:(ASN10 ASP11 ILE12 VAL14 GLN90 VAL94 GLN420 GLU422 LYS423 ALA691 SER692 ALA693)
14	11	-0.25	13	23	1:(ARG577 LEU580 GLU581 TYR644 GLU653 PHE655)
15	41	-0.26	14	21	1:(GLY305 VAL335 ASN336 MET339 SER340 PHE343 PHE373 THR374 SER375 LEU376)
16	15	-0.26	13	21	1:(LEU49 LEU50 SER53 PRO54 PRO57 THR170 ARG199 LEU718 ALA719 ASP722 TRP726)
17	15	-0.27	8	15	1:(VAL578 GLU581 ALA582 ALA617 ARG620 PHE621 TYR644)
18	19	-0.28	10	21	1:(TYR196 ILE315 GLU316 SER317 TYR318 ASN394)
19	10	-0.29	4	14	1:(GLU428 ASP430 ASP431 TYR540 LEU544 VAL603 ARG604 LEU605 ASP606)
20	16	-0.30	11	17	1:(LYS365 ASP366 LYS367 LEU369 LYS459)
21	23	-0.31	12	23	1:(GLU316 TYR318 GLU329 PHE381 PRO387 GLY389 ILE390 ASN391 ILE392 ASN394)
22	16	-0.33	8	17	1:(GLN5 LYS107 PHE109 THR112 SER668 ARG669)
23	6	-0.33	3	8	1:(TYR196 SER317 GLY323 SER324)
24	6	-0.33	3	11	1:(GLU713 THR716 GLN717 THR720)

The calculated binding affinity for the examined heterocyclic compounds and Dipeptidyl Peptidase III [pdb id: 3fvy] were -3.92 kcal/mol for compound **1**, -6.39kcal/mol for compound **2**, -6.43 kcal/mol for compound **3**, -6.25 kcal/mol for compound **4**, -6.42kcal/mol for compound **5**, -5.57 kcal/mol for compound **6**, -3.70 kcal/mol for compound **7**, -7.08 kcal/mol for compound **10** and -5.29 kcal/mol for reference compound. As displayed in Table 5, no result were generated for compound 8- Dipeptidyl Peptidase III [pdb id: 3fvy] and compound 9- Dipeptidyl Peptidase III [pdb id: 3fvy] complexes.

**Table 5:** Calculated scoring for compound 1-10 in kcal/mol.

	Scoring (kcal/mol)
1	-3.92
2	-6.39
3	-6.43
4	-6.25
5	-6.42
6	-5.57
7	-3.70
8	-
9	-
10	-7.08
Ref	-5.29

Ref: Ibuprofen

More so, to access the efficiency of the ligand to inhibit Dipeptidyl Peptidase III [pdb id: 3fvy], distance, bond type, residue, 2-dimensional configuration of the docked compound in the active site of the receptor were considered (Table 6). The calculated nonbonding interaction were 3.65, 2.65, 3.19 and 2.65 (Distance); H-acceptor, H-acceptor, Ionic and Ionic (bond type) and GLN 261, LYS 264, ARG 39 and LYS 264 (Residue) for compound **1**; 2.85, 2.85 and 3.23 (Distance); H-donor, Ionic and Ionic (bond type); GLU 344, GLU 344 and GLU 344 (Residue) for compound **2;** 3.29, 3.29, 3.95, and 3.97 (Distance); H-donor, Ionic, Ionic and Ionic (bond type); GLU 329, GLU 329, ASP 111, ASP 111 (Residue) for compound **3;** 3.16, 3.16 and 4.52 (Distance); H-donor, Ionic and pi-H (bond type); GLU 327, GLU 327 and PRO 387 (Residue) for compound **4;** 3.28, 3.26, 3.04, 2.86, 3.04, 2.86, 3.85, 3.28 and 4.19 (Distance); H-donor, H-acceptor, H-acceptor, H-acceptor, Ionic, Ionic, Ionic, Ionic and H-pi (bond type); GLU 451, LYS 629, LYS 629, LYS 365, LYS 629, LYS 365, LYS 629, GLU 451and HIS 455 (Residue) for compound **5;** 3.05, 3.15 and 3.68 (Distance); H-acceptor, H-acceptor and pi-H (bond type); ASN 10, ASN 117 and PRO 9 (Residue) for compound **7;** 2.76 and 3.11 (Distance); H-acceptor and H-acceptor (bond type); SER 375 and ASN 336 (Residue) for compound **10.**

**Table 6:** Selected non-bonding interaction and visual representation for the investigated complexes.

Ligand	Distance	Bond Type	Residue	2D Diagram
1	3.65	H-acceptor	GLN 261	
	2.65	H-acceptor	LYS 264	
	3.19	Ionic	ARG 39	
	2.65	Ionic	LYS 264	
2	2.85	H-donor	GLU 344 (A)	
	2.85	Ionic	GLU 344 (A)	
	3.23	Ionic	GLU 344 (A)	
3	3.29	H-donor	GLU 329 GLU 329	
	3.29	Ionic	ASP 111	
	3.95	Ionic	ASP 111	
	3.97	Ionic		
4	3.16	H-donor	GLU 327	
	3.16	Ionic	GLU 327	
	4.52	pi-H	PRO 387	
5	3.28	H-donor	GLU 451	
	3.26	H-acceptor	LYS 629	
	3.04	H-acceptor	LYS 629	
	2.86	H-acceptor	LYS 365	
	3.04	Ionic	LYS 629	
	2.86	Ionic	LYS 365	
	3.85	Ionic	LYS 629	
	3.28	Ionic	GLU 451	
	4.19	H-pi	HIS 455	
6	-	-	-	
7	3.05	Interaction	ASN 10	
	3.15	H-acceptor	ASN 117	
	3.68	H-acceptor	PRO 9	
		pi-H		
8	-	-	-	-
9	-	-	-	-
10	2.76	Interaction	SER 375	
	3.11	H-acceptor	ASN 336	
		H-acceptor		

Table 7, Table 8 showed the pharmacokinetic analysis of the lead compound (compound 10) and Ibuprofen (reference compound). The analysis was presented in ADMET predicted profile — classification and ADMET predicted profile — regression format. The prediction was displayed under three headings (model, result and probability) and the predicted model for absorption were Blood-Brain Barrier, Human Intestinal Absorption, Caco-2 Permeability, P-glycoprotein Substrate, P-glycoprotein Inhibitor and Renal Organic Cation Transporter; for distribution was Subcellular localization; metabolism were CYP450 2C9 Substrate, CYP450 2D6 Substrate, CYP450 3A4 Substrate, CYP450 1A2 Inhibitor, CYP450 2C9 Inhibitor, CYP450 2D6 Inhibitor, CYP450 2C19 Inhibitor, CYP450 3A4 Inhibitor and CYP Inhibitory Promiscuity and for toxicity, the Human Ether-a-go-go-Related Gene Inhibition, AMES Toxicity, Carcinogens, Fish Toxicity, Tetrahymena Pyriformis Toxicity, Honey Bee Toxicity, Biodegradation, Acute Oral Toxicity and Carcinogenicity (Three-class). Also, the factors considered for ADMET predicted profile — regression were Aqueous solubility, Caco-2 Permeability (absorption) and Rat Acute Toxicity, Fish Toxicity and Tetrahymena Pyriformis Toxicity (toxicity).

**Table 7:** Pharmacokinetic evaluation of compound 10.

ADMET Predicted Profile — Classification		
Model	Result	Probability
**Absorption**		
Blood-Brain Barrier	BBB-	0.96
Human Intestinal Absorption	HIA+	0.98
Caco-2 Permeability	Caco2+	0.88
P-glycoprotein Substrate	Substrate	0.57
P-glycoprotein Inhibitor	Non-inhibitor	0.72
	Non-inhibitor	0.74
Renal Organic Cation Transporter	Non-inhibitor	0.94
**Distribution**		
Subcellular localization	Mitochondria	0.44
**Metabolism**		
CYP450 2C9 Substrate	Substrate	0.64
CYP450 2D6 Substrate	Non-substrate	0.91
CYP450 3A4 Substrate	Non-substrate	0.73
CYP450 1A2 Inhibitor	Non-inhibitor	0.90
CYP450 2C9 Inhibitor	Inhibitor	0.90
CYP450 2D6 Inhibitor	Non-inhibitor	0.92
CYP450 2C19 Inhibitor	Non-inhibitor	0.90
CYP450 3A4 Inhibitor	Non-inhibitor	0.87
CYP Inhibitory Promiscuity	Low CYP Inhibitory Promiscuity	0.87
**Toxicity**		
Human Ether-a-go-go-Related Gene Inhibition	Weak inhibitor	0.95
	Non-inhibitor	0.83
AMES Toxicity	Non AMES toxic	0.87
Carcinogens	Non-carcinogens	0.76
Fish Toxicity	Low FHMT	0.59
Tetrahymena Pyriformis Toxicity	High TPT	0.93
Honey Bee Toxicity	Low HBT	0.81
Biodegradation	Not ready biodegradable	0.92
Acute Oral Toxicity	II	0.72
Carcinogenicity (Three-class)	Non-required	0.69

ADMET Predicted Profile — Regression		
Model	Value	Unit

**Absorption**		
Aqueous solubility	-4.09	LogS
Caco-2 Permeability	1.35	LogPapp, cm/s
**Toxicity**		
Rat Acute Toxicity	3.15	LD50, mol/kg
Fish Toxicity	1.72	pLC50, mg/L
Tetrahymena Pyriformis Toxicity	0.53	pIGC50, ug/L

**Table 8:** Pharmacokinetic evaluation of reference compound.

ADMET Predicted Profile — Classification		
Model	Result	Probability
**Absorption**		
Blood-Brain Barrier	BBB+	0.96
Human Intestinal Absorption	HIA+	0.99
Caco-2 Permeability	Caco2+	0.88
P-glycoprotein Substrate	Non-substrate	0.75
P-glycoprotein Inhibitor	Non-inhibitor	0.97
	Non-inhibitor	0.93
Renal Organic Cation Transporter	Non-inhibitor	0.93
**Distribution**		
Subcellular localization	Mitochondria	0.69
**Metabolism**		
CYP450 2C9 Substrate	Non-substrate	0.75
CYP450 2D6 Substrate	Non-substrate	0.91
CYP450 3A4 Substrate	Non-substrate	0.68
CYP450 1A2 Inhibitor	Non-inhibitor	0.90
CYP450 2C9 Inhibitor	Non-inhibitor	0.93
CYP450 2D6 Inhibitor	Non-inhibitor	0.92
CYP450 2C19 Inhibitor	Non-inhibitor	0.98
CYP450 3A4 Inhibitor	Non-inhibitor	0.96
CYP Inhibitory Promiscuity	Low CYP Inhibitory Promiscuity	0.96
**Toxicity**		
Human Ether-a-go-go-Related Gene Inhibition	Weak inhibitor	0.97
	Non-inhibitor	0.97
AMES Toxicity	Non AMES toxic	0.98
Carcinogens	Carcinogens	0.55
Fish Toxicity	High FHMT	0.94
Tetrahymena Pyriformis Toxicity	High TPT	0.99
Honey Bee Toxicity	High HBT	0.74
Biodegradation	Ready biodegradable	0.51
Acute Oral Toxicity	III	0.80
Carcinogenicity (Three-class)	Non-required	0.73

ADMET Predicted Profile — Regression		
Model	Value	Unit

**Absorption**		
Aqueous solubility	-3.90	LogS
Caco-2 Permeability	1.74	LogPapp, cm/s
**Toxicity**		
Rat Acute Toxicity	2.30	LD50, mol/kg
Fish Toxicity	1.31	pLC50, mg/L
Tetrahymena Pyriformis Toxicity	1.38	pIGC50, ug/L

## 4 Experimental Design, Materials and Methods

Ten heterocyclic compounds were modeled and optimized in three ways (selected compound; selected compound + Cadmium and selected compound + Lead) using Spartan ’14 [1,2]. These compounds were chosen based on their reported efficacy. Three selected features (HOMO, LUMO and energy gap) for each category for the entire compound under investigation were retrieved and reported. The entire compound were prepared for docking using induced fit docking method via molecular operating environment (MOE) software; while the receptor was retrieved from protein data bank Dipeptidyl Peptidase III [pdb id: 3fvy] [3] and treated by removing water molecules and small molecules (Zn, Mg and Cl) downloaded with the receptor and saved in moe format. The investigated complexes were calculated using induced fit docking method with 30 poses via molecular operating environment software [4,5]. The correlation between the calculated descriptors and the scoring for the examined complexes were executed using Microsoft excel software and the predicted squared correlation coefficient was reported accordingly. The pharmacokinetic evaluation of compound **10** and reference compound were accomplished using ADMETSar software [6,7] and the obtained outputs were reported.

## Limitations

More investigation beyond what has been presented is expected so as to further probe the biological role of the investigated compounds.

## Ethics Statement

This study does not involve studies with animals and humans.

## CRediT authorship contribution statement

**Kehinde Adeola Bolaji:** Conceptualization, Methodology, Data curation, Writing – original draft, Visualization, Investigation, Writing – review & editing. **Abel Kolawole Oyebamiji:** Conceptualization, Methodology, Data curation, Writing – original draft, Visualization, Investigation, Writing – review & editing. **Godwin Oladele Olutona:** Conceptualization, Methodology, Data curation, Writing – original draft, Visualization, Investigation, Writing – review & editing.

## Data Availability

Mendeley DataDataset on Effect of Cadmium and Lead on Chemical Reactivity of Selected Heterocyclic Compounds as Potential Dipeptidyl Peptidase III Inhibitors Using Insilico method (Original data).
